# Mutations in the coat complex II component SEC23B promote colorectal cancer metastasis

**DOI:** 10.1038/s41419-020-2358-7

**Published:** 2020-03-02

**Authors:** Chunyuan Yang, Nan Chen, Xiang Li, Dan Lu, Zhiyuan Hou, Yuhua Li, Yan Jin, Jin Gu, Yuxin Yin

**Affiliations:** 1 Department of Pathology, School of Basic Medical Sciences, Peking University Health Science Center, 100191 Beijing, P. R. China; 2 Department of Gastrointestinal Center, Peking University Cancer Hospital and Institute, 100142 Beijing, P. R. China; 3 Institute of Systems Biomedicine, School of Basic Medical Sciences, Beijing Key Laboratory of Tumor Systems Biology, Peking-Tsinghua Center for Life Sciences, Peking University Health Science Center, 100191 Beijing, P. R. China

**Keywords:** Metastasis, Cancer genetics, Metastasis, Colorectal cancer, Genetics research

## Abstract

Metastasis is the leading cause of death for colorectal cancer (CRC). However, the protein transport process involved in CRC metastasis remains unclear. In this report, we use whole-exome sequencing and bioinformatics analysis to identify somatic mutations in CRC samples and found mutations of the protein transport gene Sec23 homolog B (*SEC23B*) in patients with metachronous liver metastasis. We show that deletion of SEC23B suppresses the membrane localization of adhesion proteins and augments cell mobility. SEC23B mutations either cause a premature stop (C649T) or impair its protein transport activity (C1467G and T488C + G791A + G2153A). Furthermore, SEC23B mutations inhibit the transport of epithelial cell adhesion molecule (EPCAM) and CD9 molecule, thereby attenuating cell adhesion and promoting invasiveness both in vitro and in vivo. Taken together, these data demonstrate the important impact of *SEC23B* mutations on metastasis, and we propose that SEC23B is a potential suppressor of CRC metastasis.

## Introduction

Colorectal cancer (CRC) is one of the most prevalent cancers worldwide. According to the World Health Organization (WHO), nearly 1.1 million individuals are diagnosed with CRC each year^1^. More than half of CRC patients develop metastatic lesions, and liver is the most frequently involved organ^2,3^. In general, CRC liver metastasis is divided into synchronous liver metastasis found within 6 months of diagnosis, and metachronous liver metastasis (MLM) found later than six months after diagnosis. Although there have been numerous studies on the mechanisms of CRC synchronous metastasis using whole-exome sequencing^4–7^, the mechanisms underlying MLM seem lacking.

Metastasis is a sequential, multi-step pathological process, which requires cooperation of proteins inside and outside of cells^8,9^. In eukaryotes, the protein secretory pathway consists of a set of organelles and cargo-bearing vesicles^10,11^. The first step of this pathway is the transport of protein from endoplasmic reticulum (ER) to Golgi apparatus, mediated by the coat protein II (COPII) complex, which is composed of secretion associated Ras related GTPase 1 (SAR1), SEC23-SEC24 heterodimeric complex, and SEC13-SEC31 heterodimeric complex^12^.

SEC23B is one of the homologues of SEC23 in mammals, which functions in COPII assembly and vesicle budding^13–15^. Knockout of *Sec23b* is lethal in mice as it results in pancreatic insufficiency and hypoglycaemia^13^. Mutations of *SEC23B* have been found to be the cause of type II Congenital Dyserythropoietic Anemia (CDAII)^16^ and Cowden Syndrome^17^. However, the impact of SEC23B on tumor metastasis is largely unknown.

Here, we have identified *SEC23B* mutations in primary CRC samples which give rise to MLM. We demonstrate that dysregulation of protein transport due to SEC23B mutation leads to remodeling of extracellular matrix and enhanced metastatic capacity. Our study highlights the significance of SEC23B in tumor metastasis, and provides a potential biomarker for CRC progression.

## Materials and methods

### Whole-exome sequencing of tumor samples

Genomic DNA was extracted from matched tumor tissues and non-neoplastic tissues adjacent to the tumor using the standard protocol for the QIAGEN DNase Blood and Tissue kit (QIAGEN, Hilden, Germany). All samples were quality controlled for purity using a NanoDrop spectrophotometer and Qubit Fluorometer (Thermo Fisher Scientific Inc., Waltham, MA, USA). DNA was captured on an Agilent SureSelect DNA library construction (SureSelect V3, Palo Alto, CA, USA). Tumor samples of S3, S6, S8, S11, S12, S16, S19, S20, S21 were sequenced on the Illumina Hiseq1000 instrument (Illumina, Inc., San Diego, CA, USA), and the remaining tumor samples as well as all the normal samples were sequenced on the Illumina HiseqX10 instrument (Illumina, Inc., San Diego, CA, USA), as recommended in the manufacturer protocols for paired 100-bp reads. Image analysis and base calling were performed using the Illumina pipeline with default parameters.

### Somatic mutation detecting and functional evaluations

Reads were aligned to the human reference hg19 genome assembly using Burrows-Wheeler Aligner (BWA; http://bio-bwa.sourceforge.net/), and duplicated read pairs were removed. Somatic mutations were called with Genome Analysis Toolkit (GATK)^18^ (https://software.broadinstitute.org/gatk/) best practice pipeline and Mutect^19^. Variants were annotated using ANNOVAR^20^ (http://www.openbioinformatics.org/annovar/). Mutations between groups were compared with MutSigCV^21^ (http://software.broadinstitute.org/cancer/software/genepattern/modules/docs/mutsigcv) and IntOGen-OncodriveFM^22^ (https://www.intogen.org/search). The effects of the identified variants were assessed using Sorting Intolerant Form Tolerant (SIFT)^23^ (http://sift.jcvi.org), and Polymorphism Phenotyping v2 (PolyPhen-2)^24^ (http://genetics.bwh.harvard.edu/pph2). Conservation analysis was performed using MEGA7 (Molecular Evolutionary Genetics Analysis) and MUSCLE (Multiple Sequence Alignment).

### Cell culture, antibodies, reagents, and mice

SW480, SW620, HCT116, DLD1, LOVO, HT29, HEK293, and B16 cell lines were obtained from the ATCC and recently been authenticated by STR profiling. The SW480 cell line was cultured in RPMI 1640 (Corning) supplemented with 10% FBS (PAN, P30-3302), and the SW620, HCT116, DLD1, LOVO, HT29, HEK293 as well as B16 cell lines were cultured in DMEM (Corning) supplemented with 10% FBS. These cell lines were cultured in a 37 °C incubator with 5% (v/v) CO_2_.

Mouse monoclonal anti-SEC23B antibody was generated against the synthetic peptide LTKPAMPMQQARPAQPQEHP, and was validated in our laboratory (Supplementary Fig. 1a). The commercial antibodies used in this study included GFP (RM1008), GAPDH (RM2002), and α-Tubulin (RM2007) antibodies from Sungene Biotech; EPCAM (66316-1-AP), E-cadherin (20874-1-AP) and PDI (11245-1-AP) from Proteintech; FLAG (M2-3165) from Sigma-Aldrich; GM130 (A5344) from ABclonal; CD9 (sc13118) from Santa Cruz.

The reagents used in this paper included Fibronectin (354008) from Biocoat, Matrigel (356234) from BD, puromycin (sc-205821A) from Santa Cruz, MG132 (Carbobenzoxy-L-leucyl- L-leucyl-L-leucinal) (MB5137) from EPSILON, and chlorhexidine (CHX) (C7698) from SBJBIO, anti-fade mounting reagent (C1210) from Polygen.

C57BL/6N was purchased from Charles River Laboratories.

### Plasmids

pCMV-tag-2b (Addgene) and pEGFP-N1 (Addgene) were used to overexpress wild-type (WT) or mutant SEC23B. The pX330-U6-Chimeric_BB-CBh-hSpCas9 (Addgene) was used to knockout SEC23B. The pCDH-CMV-MCS-EF1 lentiviral system (Addgene) was used for WT and mutant SEC23B expression. The Str-Ii_VSVG-SBP-mCherry (Addgene, #65301) was used for RUSH assay.

### Lentiviral infections

Clustered regularly interspaced short palindromic repeats (CRISPR) knockout of SEC23B in SW480 cells was mediated by transfection of lentivirus targeting mock (ATCGACTAGCCACTCAGAC) or SEC23B (5′-GGAACGTGTGGCCTTCCAGC-3′). To express WT or mutant SEC23B, cells were infected with the pCDH-CMV-MCS-EF1 lentiviral overexpression system. After harvest from the HEK293 medium, the lentivirus was added to the target cell line followed by puromycin selection.

### Protein production rate and protein stability detection

SEC23B depleted SW480 cells expressed with GFP, GFP-tagged WT, M1, or M3 mutations were treated with 100 µg/ml MG132, and collected at 0, 12 or 24 h independently to evaluate protein production rate. SEC23B depleted SW480 cells expressed with GFP, GFP-tagged WT, M1, or M3 mutations were treated with 100 µg/ml CHX, and collected at 0, 4, 8 or 12 h independently to evaluate protein stability.

### Flag pull-down assay

SW480 cells were transfected with pCMV empty plasmid, pCMV-WT or mutant SEC23B. 24 h after transfection, cells were harvested and homogenized in lysis buffer (50 mM Tris pH 7.5, 150 mM NaCl, 2 mM EDTA, 0.5% NP40 and 1 mM PMSF). Equal amounts of protein in different groups were incubated with anti-FLAG M2 Affinity Gel (Sigma Aldrich) for 4 h. Protein-beads complexes were washed with lysis buffer. Proteins were loaded into NuPAGE 4–12% gels (Invitrogen) and visualized with silver staining (Pierce Silver Stain Kit). The potential interaction proteins were analyzed with mass spectrometry (MS) (MS Analysis was performed as previously reported^25^).

### Immunofluorescence (IF) and confocal microscopy

Cells cultured on glass slides were washed twice with PBS, and then fixed with cold acetone for 15 min. After washing for 20 min with PBS and subsequent permeabilization with 0.5% Triton-X100, cells were blocked with 1% BSA (Bovine Serum Albumin) and immunoblotted with antibodies against PDI, GM130 or EPCAM overnight at 4 °C. The cells were then incubated with secondary antibodies (Invitrogen) at room temperature for 1 h and stained with 0.5 µg/ml DAPI for 5 min. After mounting with anti-fade mounting reagent, images were acquired with a Nikon TCS A1 confocal microscope and analyzed with ImageJ.

### MTT assay

After a 1-, 2- or 3-day incubation period, 20 μl of 5 mg/ml 3-(4,5-dimethyl-2-thiazolyl)-2,5-diphenyl-2-H-tetrazolium bromide (MTT) solution was added to each well and the plate was then incubated at 37 °C for 4 h. After the dissolve of formazan crystals, the absorbance was determined spectrophotometrically at 570 nm. MTT assays were performed in triplicate.

### RUSH assay

The retention using selective hooks (RUSH) assay was performed as previously reported with some modifications^26^. To perform the live cell imaging, cells were seeded into a glass-bottom cell culture dish 2 days before acquisition. Transfect cells with Str-Ii_VSVG-SBP-mCherry plasmid followed by 24-h incubation at 37 °C. Cell culture medium was exchanged to 40 μM biotin-containing medium, and photos were taken 0 min, 15 min, and 1 h later.

### Cell adhesion assay

To test the cell adhesion capacity of cells with WT or mutant SEC23B, fibronectin was diluted to 10 μg/ml with RPMI 1640, then coated onto 24-well cell culture plates (Corning) for 1 h at 37 °C. 5 × 10^4^ cells with WT or mutant SEC23B were seeded into each well and cultured at 37 °C. Two hours later, cells were washed with PBS and fixed with cold methyl alcohol, and then photographed and counted with ImageJ. Cell adhesion assays were performed in triplicate.

### Cell migration and invasion assay

For cell migration assay, SW480 cells expressing WT or mutant SEC23B were seeded into the upper chamber of a transwell (Corning, 8-μm pore size) coated with fibronectin. The upper chamber contained RPMI 1640 with 2% BSA. The bottom chamber contained RPMI 1640 with 10% FBS. After an 18-h incubation, cells remaining in the upper chamber were removed, and those attached to the bottom surface were stained with crystal violet and photographed.

For cell invasion assay, cells were prepared as in the migration assay, except that the transwell chamber was coated with Matrigel and incubation time was extended to 36 h. All assays were performed in triplicate.

### B16 mouse metastasis model

The pCDH-CMV-MCS-EF1 lentiviral system was used to stably overexpress WT (*n* = 12), M1 mutant (*n* = 12) or M3 mutant (*n* = 12) SEC23B in B16 mice melanoma cells. After puromycin selection, cells were injected into the tail vein of 8-week-old male C57BL/6N mice (mice were random chosen for this experiment), at 8 × 10^5^ cells/mouse in 100 μl PBS. Two weeks later, all mice were sacrificed and lung were excised and fixed in 4% paraformaldehyde. Lung architecture was evaluated with standard H&E stains. Single-blind method was used in this trial.

### Statistics

Mutation numbers were considered only in samples with at least one somatic mutation. Comparison between the mutation number of TCGA data and our mutation data was done with *T* test (*p* = 0.1106, the alternative hypothesis was that the mutation number of our samples was greater than those used in the TCGA). Comparison between the somatic mutation distribution of TCGA data and our somatic mutation was done with Kolmogorov-Smirnov test (*p* = 0.9831). R Software did the Kolmogorov-Smirnov test with the default set between study samples and TCGA data.

Top 20 mutated genes in CRC were found on Catalogue Of Somatic Mutations In Cancer (COSMIC)^27^ (https://cancer.sanger.ac.uk/cosmic). Tissue selection were performed on the COSMIC Cancer Browser and the arguments were set to “Large intestine”, “Colon”, “Include all”, “Include all”.

Self-script was used to count mutations that led to amino acid alteration. Based on annotation, only non-synonymous mutations and stop-gain mutations in the exome were considered. Genes showed mutations in more than one MLM patient but no mutation in controls were selected as candidates for further investigation.

Statistical analysis of western blot experiments were based on results of three independent experiments. The greyscale of each band was measured by Photoshop. The relative expression of proteins in GFP, SEC23B M1, or SEC23B M3 group compared with SEC23B group was calculated by dividing each greyscale against the greyscale in SEC23B group.

All statistical data are presented as the mean ± standard deviation (SD). Significance of difference between two groups was evaluated by unpaired, two-tailed Student’s T test, Two-way ANOVA, Kolmogorov-Smirnov test, and Log-rank test where appropriate, using GraphPad Prism. A *P* value of <0.05 was used as a cutoff for statistical significance.

### Data acquisition

Representative images of cell adhesion assay, cell migration assay and cell invasion assay were acquired by an Olympus microscope. Representative images of IF experiments were acquired by a Nikon TCS A1 confocal microscope and analyzed with ImageJ. Representative photos of B16 colonies in lung were acquired by a Canon camera.

### Study approval

Human colorectal specimens were obtained from primary resected tumors and their adjacent non-neoplastic colon tissues during CRC in situ resection surgery performed at Beijing Cancer Hospital (Beijing, P.R. China) under a research agreement (No. 2015KT33). All patients agreed with the use of their resected tissues for research purposes. The study was approved by the Ethics Review Committees of Peking University Cancer Hospital & Institute. All animal studies were performed according to the ethical standards of the National Institutes of Health Animal Use Guidelines and under approval by the Peking University Biomedical Ethics Committee (No. LA2018032).

## Results

### Clinical cohort and whole-exome sequencing of cancer samples

**Fig. 1:**
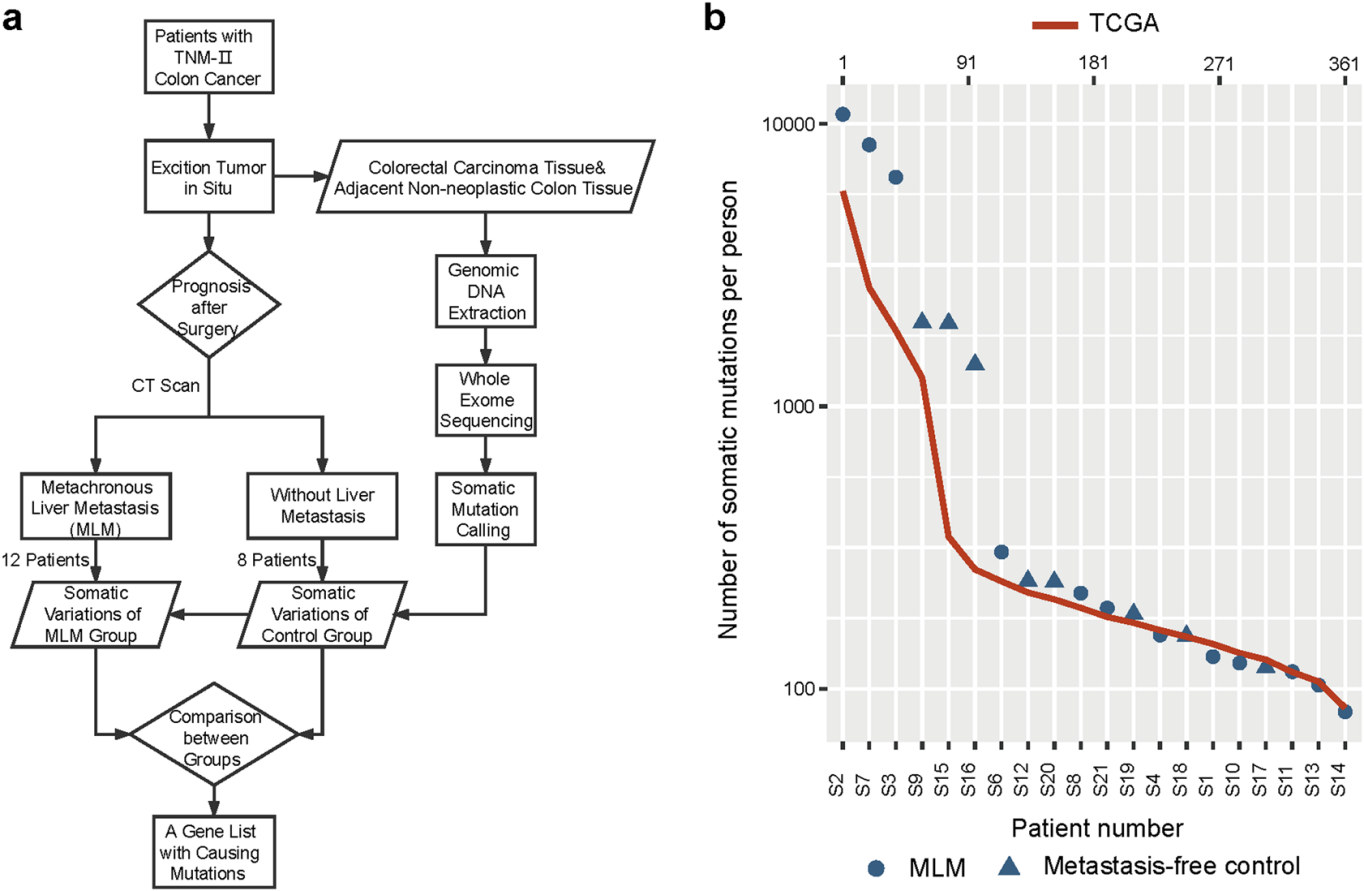
Workflow of the study. **a** Twenty patients enrolled in this study include 12 MLM patients and 8 metastasis-free controls. The workflow is divided into four main steps. Step 1: excision of TNM stage II CRC to obtain tumor samples and the adjacent normal tissues. Step 2: CT scan to look for metastases six months after resection of primary CRC. Samples are divided into the MLM group and the metastasis-free control group according to the CT result. Step 3: whole-exome sequencing on paired tumor and the adjacent normal tissue to find somatic mutations. Step 4: screening and comparison of mutations with multiple strategies. **b** Somatic mutation number and distribution in the study. The upper horizontal axis shows the arrangement of 361 patients from the TCGA database (red line). The bottom horizontal axis shows the arrangement of patients from the MLM group (blue circle) and the metastasis-free control group (blue triangle). The vertical axis shows the total somatic mutation number of each CRC patient. Our data show similar somatic mutation number and distribution with the TCGA data (*T* test, *p* = 0.5048).

To identify mutations associated with CRC metastasis, we chose patients who were at the pathologic Tumor Node Metastasis (TNM) stage II (based on the seventh edition of the American Joint Committee on Cancer (AJCC) TNM staging system^28^). These patients had no evidence of synchronous liver metastasis by computed tomography (CT) scan and no pathologic evidence of positive local/regional lymph nodes in the resected CRC specimen. After evaluation of information regarding diagnosis and prognosis, 20 of these patients were selected. Of note, we took tissues from primary resected tumors of each patient for whole-exome sequencing and further investigation (Fig. 1a), and their adjacent non-neoplastic colon tissues were used for comparison. Six months after CRC resection, patients were reevaluated for metastases using CT scan. Twelve patients who developed metastases in the liver formed the MLM group. The remaining eight patients who had no CT evidence of tumor metastasis over 1.5 years formed the metastasis-free control group.

Whole-exome sequencing was carried out on both primary tumor and the adjacent colorectal tissue in each case, while metachronous liver metastases were not subjected to the sequencing analysis. The average sequence depth was over 50 independent reads per targeted base, and this sequencing covered 98% of the exome area. We used the GATK standard best practice pipeline and Mutect to identify somatic single nucleotide substitution. We also used ANNOVAR to annotate variants and functional mutation information. To be specific, only somatic mutations from the exome were taken into consideration in this study, and the result showed that the average number of somatic single nucleotide substitution in these 20 tumor samples was 1669.8.

To assess the reliability of our sequencing results, we compared our data with TCGA CRC project^4^. There was no significant difference in mutation number or distribution between our samples and the TCGA data (Fig. 1b). These results indicate the high quality of sequencing and somatic mutation detection in our study, and suggest that our results are reliably representative of CRC sequencing data.

### Strategies for screening mutations with functional influence

In this report, we posited that amino acid substitution caused by gene mutations was likely a contributing factor in CRC metastasis. Meanwhile, genes specifically mutated in tumors from MLM patients would be expected to retain their functions in patients of the metastasis-free control group. Mutations in the MLM and metastasis-free control group were compared with strategies as follows.

**Fig. 2:**
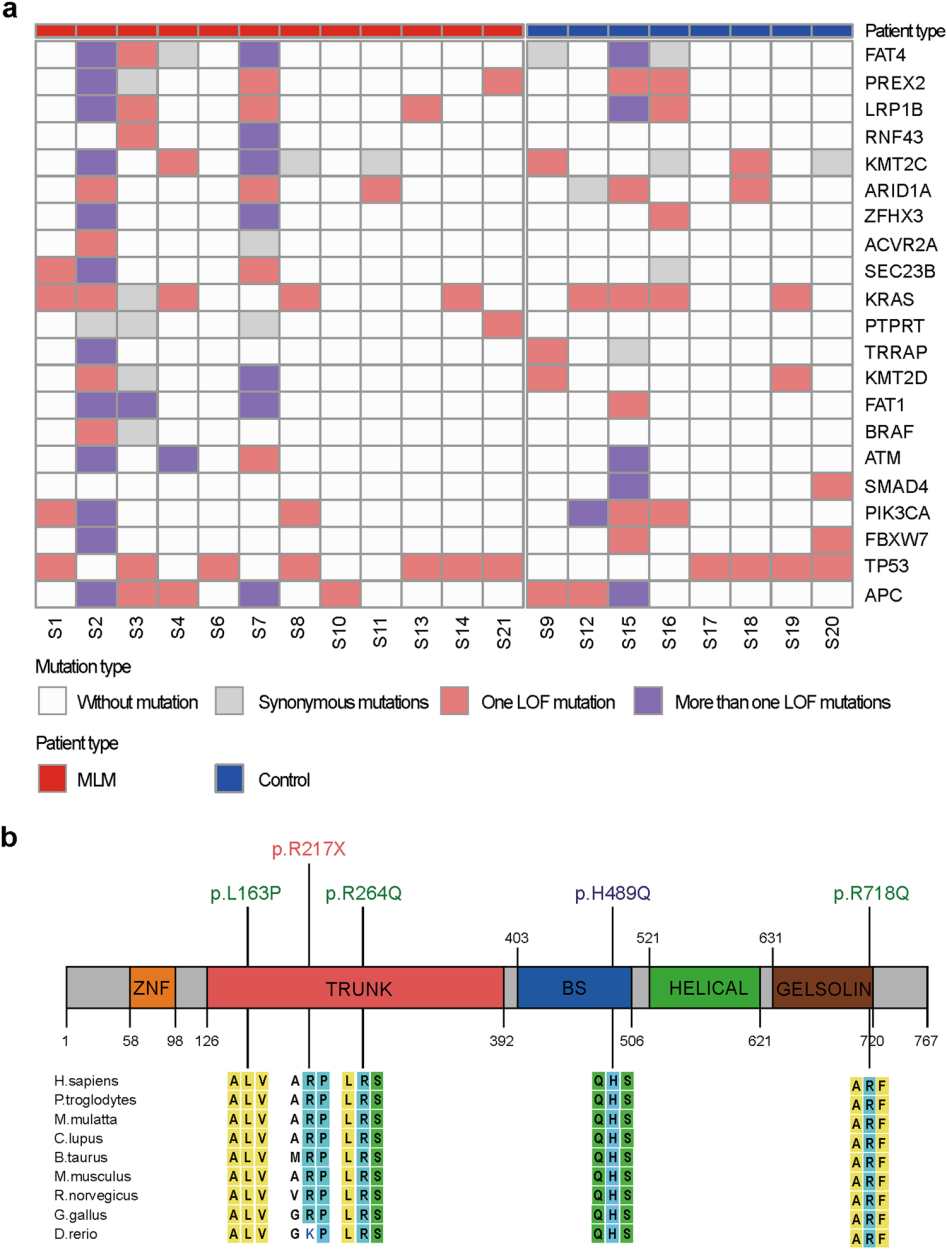
Identification of SEC23B mutations. **a** Distribution of mutations of 20 most frequently mutated genes and SEC23B in all samples. Columns represent samples and rows represent genes. White blocks represent samples with no mutation in the SEC23B gene. Gray blocks represent samples with synonymous mutations in the SEC23B gene. Pink blocks represent samples with only one mutation in the gene. Purple blocks represent samples with two or more mutations in the gene. There are no significant differences in mutation frequency of these 20 genes between MLM group and control group, but the mutation frequency of SEC23B is significantly larger in the MLM group. **b** Location and homology of SEC23B mutations identified in the MLM group. Top: locations of SEC23B mutations in patient S1 (purple), S2 (green), and S7 (pink). Bottom: the sites of SEC23B mutations are evolutionarily conserved among species.

Twenty genes, which are most frequently mutated in CRC in the COSMIC database, were first evaluated in our samples, and there was no significant difference in mutation number between the MLM group and the control group (Fig. 2a). It is worth to mention that the mutation frequency of APC regulator of WNT signaling pathway (*APC*) identified in our study was lower than reported^4^, and one possible reason is that frame-shift mutations were not selected in our whole-exome sequencing analysis results.

Next, MutSigCV was used to analyze the mutation burden in these cancer samples. This revealed a single gene designated tumor protein p53 (*TP53*) in the MLM samples that exhibited more mutations (*TP53* mutations were found in seven MLM samples and four control samples, *p* = 0.0086). Since TP53 is primarily recognized as a suppressor for tumor initiation^29^, analysis of other metastasis-related genes is warranted.

As the final step, we used OncodriveFM to help determine the significance of the mutated genes in our tumor samples. This allowed calculation of the functional impact of each non-synonymous single nucleotide variant in the tumor samples. Based on the supposition that the driver mutations of interest were mutations that change the coding of proteins together with the result of OncodriveFM, a search of all somatic mutations yielded a list of eligible genes, and *SEC23B* was one of these genes (Supplementary Table 1).

### *SEC23B* mutations occur in evolutionally conserved sites

There were five *SEC23B* mutations, namely T488C, C649T, G791A, C1467G, and G2153A, in tumors from three patients in the MLM group (Fig. 2b, upper row). The C649T mutation found in patient S1 was a nonsense mutation. Three mutations T488C, G791A and G2153A found in patient S2 and the C1467G mutation found in patient S7 were missense mutations. Among these mutations, the C649T and G791A mutations were reported in two independent endometrial cancer cases, the G2153A mutation was identified in a case of liver cancer^17,30^, while the other mutations have not been reported in the COSMIC database.

SEC23B mutations in patients with MLM were located in the trunk domain, the beta-sandwich domain, and the C-terminal gelsolin-like domain^12^ (Fig. 2b, upper row). These mutations sites were evolutionarily conserved, especially among mammals (Fig. 2b, lower row and Supplementary Table 2). Moreover, all of these mutations were predicted to be disease-causing mutations by PolyPhen-2 and SIFT (Supplementary Table 3). These results indicate that the SEC23B mutations are clinically significant.

### Deletion of SEC23B promotes tumor metastasis

**Fig. 3:**
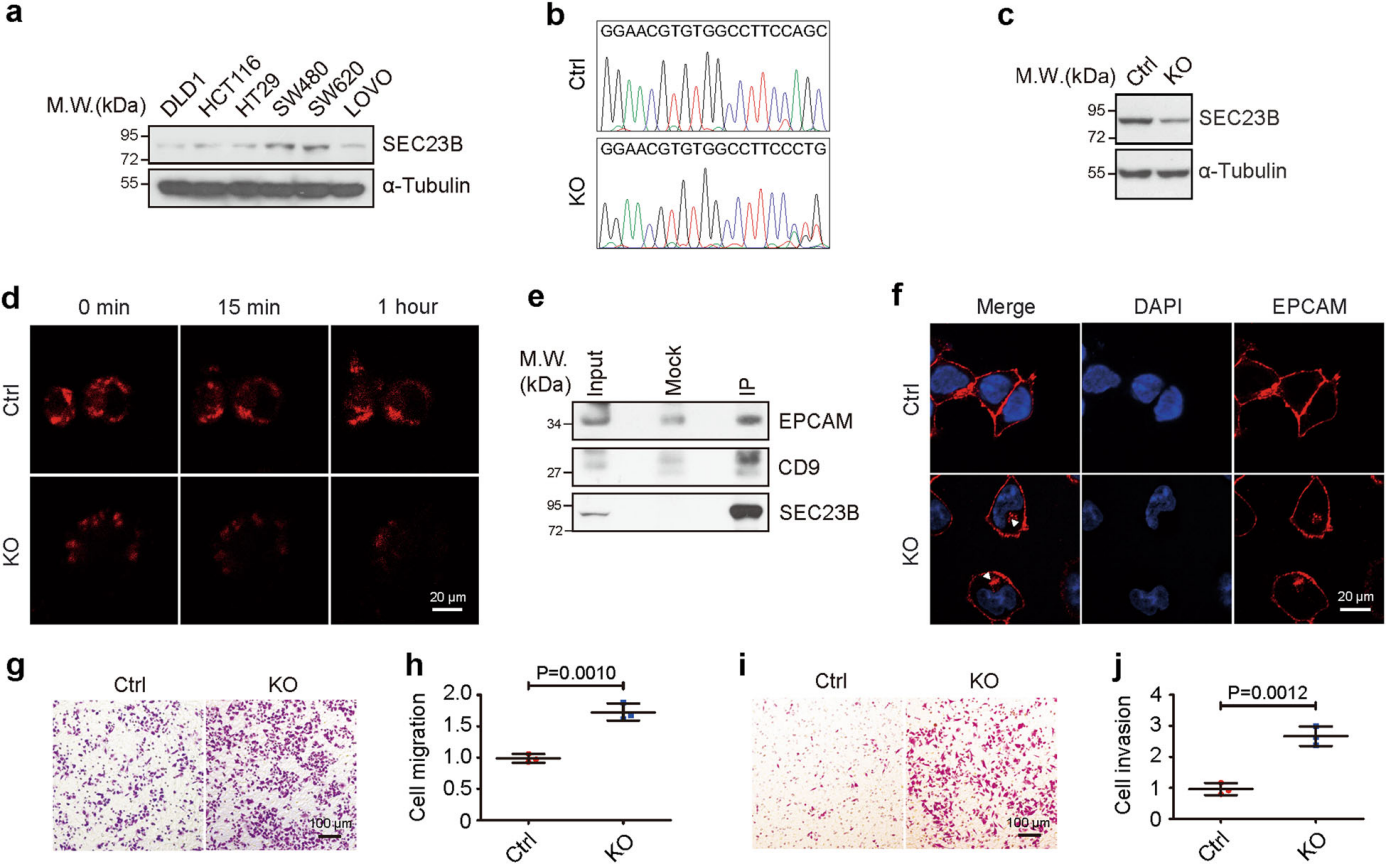
Down-regulation of SEC23B inhibits protein transport and promotes cell mobility. **a** Western blot analysis of SEC23B expression in DLD1, HCT116, HT29, SW480, SW620, and LOVO cell lines. Cells were harvested and immunoblotted with antibodies against SEC23B and α-Tubulin. SW480 and SW620 exhibit higher levels of SEC23B expression than the other cell lines (lanes 4 and 5 vs. lanes 1, 2, 3, and 6). α-Tubulin was used as loading control. **b** Validation of CRISPR knockout of SEC23B. Schematic illustration of CRISPR target region and sequence results of control (Ctrl) and SEC23B heterozygous knockout cell line (KO). **c** Western blot analysis of SEC23B expression in Ctrl cell and KO cell. α-Tubulin was used as loading control. **d** Live cell imaging of Ctrl cell and KO cell expressing streptavidin-KDEL and VSVG-SBP-mCherry (red). Pictures were taken at 0, 15 min, and 1 h after biotin treatment. Cells depleted of SEC23B show inhibited protein transport activity. Scale bars: 20 μm. **e** Interaction of SEC23B with EPCAM and CD9. SW480 cell was homogenized and immunoprecipitated with homemade monoclonal antibody against SEC23B, and immunoblotted with antibodies against EPCAM or CD9. **f** Distribution of EPCAM (red) in Ctrl cell and KO cell. Cell nuclei are shown with DAPI staining (blue). White arrows indicate the position of EPCAM in the cell plasma. Scale bars: 20 μm. **g**, **h** Representative images (**g**) and statistical analysis (three independent experiments) (**h**) of migration assay. Deletion of SEC23B promotes cell migration (*T* test). Scale bars: 100 μm. **i**, **j** Representative images (**i**) and statistical analysis (three independent experiments) (**j**) of invasion assay. Deletion of SEC23B promotes cell invasion (T test). Scale bars: 100 μm.

To investigate the impact of SEC23B on tumor metastasis, we evaluated SEC23B expression in several colon cancer cell lines. SW480 cells and their metastatic counterpart SW620 cells had relatively higher levels of SEC23B expression than the other cell lines (Fig. 3a). To determine whether SEC23B mutations promote tumor metastasis, SW480 was selected as a representative cell line, and SW480 cells were used in all experiments unless otherwise noted.

To assess the function of SEC23B on tumor progression, we established a *SEC23B*-knockout (KO) cell line using CRISPR technique, and validated the down-regulation of SEC23B with sequencing (Fig. 3b) and immunoblotting (Fig. 3c). Of note, we only got the heterozygous knockout cell line instead of homozygous knockout cell line, presumably because fully knockout of *SEC23B* was lethal. The proliferation rate of *SEC23B* knockout cell was not significantly changed in MTT assay (Supplementary Fig. 1b), suggesting that tumor metastases in patient with C649T mutation were unlikely due to excessive primary tumor growth.

Since SEC23B functions in protein transport, we used the RUSH system^31^ to detect protein transport efficiency in *SEC23B* deleted cells, and we observed delayed protein transport in cells knockout of *SEC23B* compared with the control cells (Fig. 3d). To identify the major components affected by *SEC23B* loss, we analyzed the proteins in the membrane of cells knockout of *SEC23B* and the control cell with MS (Supplementary Table 4). The result revealed an alteration of proteins involved in cell adhesion. To validate the dysfunctional protein transport process in *SEC23B* knockout cell, we focused on two genes that significantly altered in MS results, namely epithelial cell adhesion molecule (*EPCAM*) and *CD9*. EPCAM belongs to the cell adhesion molecule (CAM) family and participants in cell adhesion^32^. CD9 is a tetraspanin family member, and it regulates cell adhesion and mobility^33^. Both molecules play important roles in tumor metastasis^32,34^. We first examined the interaction of SEC23B with EPCAM and CD9, and found that SEC23B was able to interact with both EPCAM and CD9 (Fig. 3e), suggesting that SEC23B was involved in the transport of EPCAM and CD9. Next, IF was used to detect the distribution of EPCAM. The result showed retention of EPCAM (Fig. 3f) in the cytoplasm of cells deficient of *SEC23B*, indicating impaired protein transport due to *SEC23B* deficiency. Furthermore, *SEC23B* deletion promoted cell migration (Fig. 3g, h) and invasion (Fig. 3i, j) in transwell assay. Collectively, these results argue that down-regulation of SEC23B expression inhibits protein transport and augments cell mobility.

### C1467G and T488C + G791A + G2153A mutation of SEC23B impair protein stability and localization

**Fig. 4:**
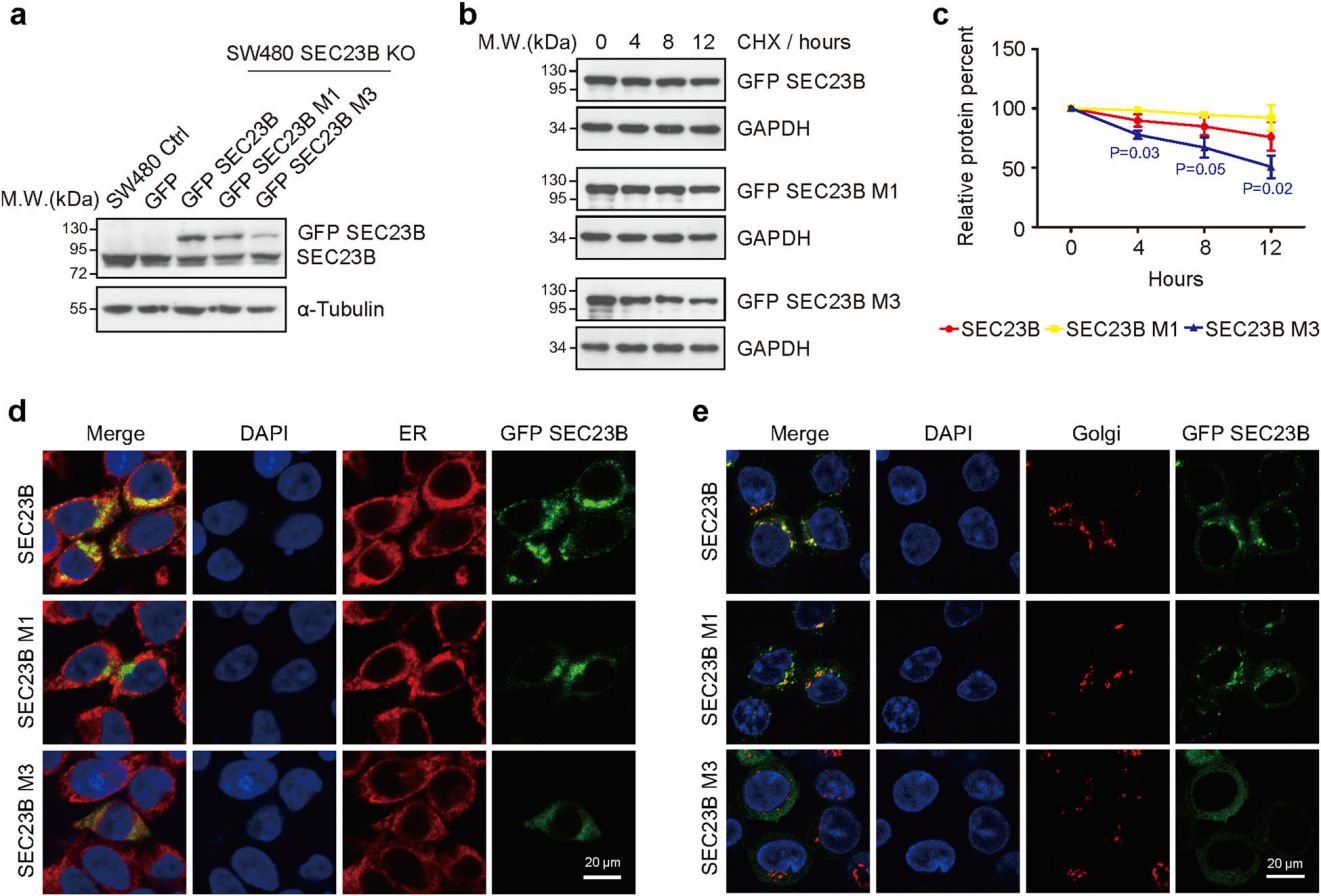
The M1 and M3 mutations of SEC23B lead to protein instability and abnormal distribution. **a** Expression of GFP, GFP-tagged WT, M1 and M3 mutations of SEC23B in SEC23B depleted cells. α-Tubulin was used as loading control. **b** Half-life analysis of WT (upper row), M1 (middle row), and M3 (bottom row) mutation of SEC23B. SW480 cells expressing WT, M1, and M3 SEC23B were treated with CHX, and collected at 0, 4, 8 or 12 h, respectively. GAPDH was used as loading control. **c** Quantitative analysis of the half-life of SEC23B (three independent experiments, *T* test). **d**, **e** Colocalization of GFP-tagged SEC23B (green) with ER (red) (**d**) or Golgi apparatus (red) (**e**) measured by IF. M1 and M3 mutations decrease the colocalization level of SEC23B with ER or Golgi apparatus. Merged areas of localization are shown in yellow. Scale bars: 20 μm.

To better recapitulate the heterozygous mutations of *SEC23B* found in the patient samples, we established cell lines expressing GFP protein, GFP-tagged WT SEC23B, GFP-tagged C1467G or T488C + G791A + G2153A mutant of SEC23B in *SEC23B*-knockout SW480 cell line (Fig. 4a). For the sake of brevity, in the following text, “M1” was used to represent the C1467G mutation in patient S7, and “M3” was used to represent the T488C + G791A + G2153A mutation in patient S2.

Evaluation of protein expression showed that the protein level of M3 mutation was significantly lower than WT protein (Fig. 4a). To investigate the underlying mechanisms, we assessed protein production rate with the proteasome inhibitor MG132, and found no significant difference in cells with WT SEC23B and M1 or M3 mutations (Supplementary Fig. 2). The protein synthesis inhibitor CHX was then used to detect protein stability (Fig. 4b), and the result showed that the half-life of M3 mutant protein was much shorter than that of WT SEC23B (Fig. 4c), indicating that M3 mutation destabilized SEC23B protein.

Then, we used IF to detect the distribution of mutant SEC23B protein (Fig. 4d, e). WT SEC23B distributed in cytoplasmic foci (Fig. 4d, e, upper row) as previously reported^17,35^. M1 mutant protein showed similar distribution pattern (Fig. 4d, e, middle row). However, M3 mutant protein was dispersed in the cytoplasm and showed no obvious foci (Fig. 4d, e, bottom row). Moreover, WT SEC23B co-localized with ER and Golgi apparatus, while the M1 and M3 mutant protein showed decreased level of co-localization with ER or Golgi apparatus. The abnormal distribution of mutant proteins strongly suggest that M1 and M3 mutations impair the function of SEC23B.

### SEC23B mutations suppress protein transport and impair distribution of proteins in extracellular matrix

**Fig. 5:**
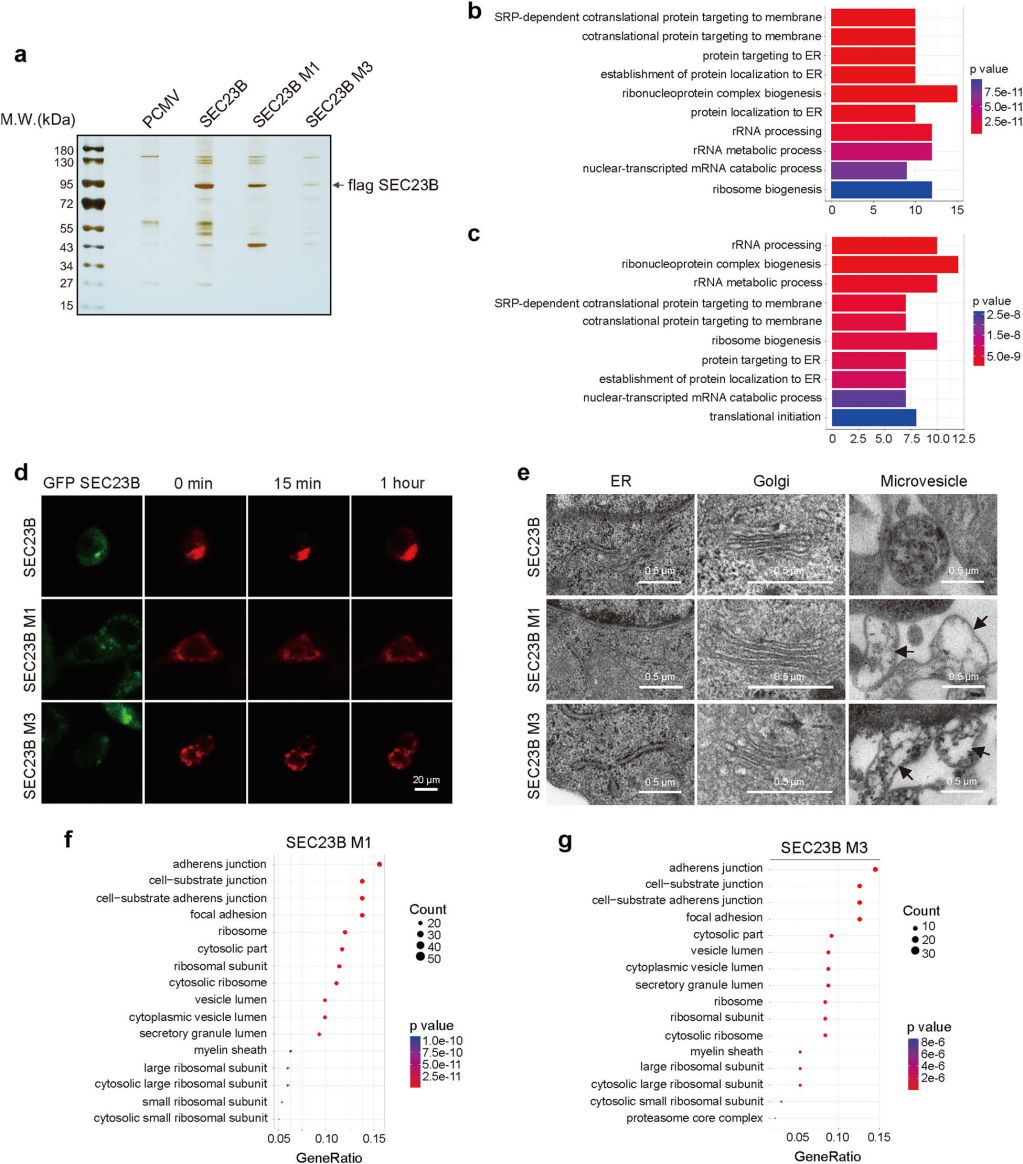
The M1 and M3 mutations of SEC23B impair protein transport. **a** Flag pulldown assay of WT or mutant SEC23B. **b**, **c** GO analysis of decreased SEC23B binding proteins upon M1 (**b**) or M3 (**c**) mutation. M1 and M3 mutations decrease the transport of cell membrane proteins. **d** Live cell imaging of cells expressing WT, M1, or M3 mutation of SEC23B (green). Cells were expressed with streptavidin-KDEL and VSVG-SBP-mCherry (red). Pictures were taken at 0 min, 15 min, and 1 hour after biotin treatment. Cells depleted of SEC23B show inhibited protein transport activity. Scale bars: 20 μm. **e** Representative TEM images of ER (left column), Golgi (middle column), and MVs (right column) morphology in cells with WT or mutant SEC23B. Black arrows indicate the locations of MVs with low electronic density. Scale bars: 0.5 μm. **f**, **g** GO analysis of down-regulated proteins in MVs of cells with M1 (**f**) or M3 (**g**) mutation reveals over-representation of cell adhesion proteins.

To identify the specific pathways affected by M1 or M3 mutation, we performed flag pull-down analysis of WT SEC23B and mutations individually (Fig. 5a). The M1 and M3 mutations decreased the binding affinity of SEC23B with a large number of proteins, as detected by MS analysis (Supplementary Table 5 and Supplementary Fig. 3a). Gene ontology (GO) analysis of proteins that were significantly changed in the MS results of M1 mutant cells revealed an enrichment of proteins involved in protein transport pathways, especially “cotranslational protein targeting to membrane” (Fig. 5b). Similar results were found in M3 mutant cells (Fig. 5c). Furthermore, we also used the RUSH system to monitor the protein transport process in cells with WT, M1, and M3 mutation of SEC23B (Fig. 5d). The mCherry-tagged VSVG transport to cell membrane within 1 h in cells with WT SEC23B, however, the transport process was delayed in cells with M1 or M3 mutation. These results indicate that M1 and M3 mutants disrupt the protein transport process.

To better characterize the abnormal protein distribution in cells with M1 or M3 mutations of SEC23B, we observed the morphology of cells with transmission electron microscope (TEM). As shown in Fig. 5e, ER (left column) and Golgi (middle column) of cells with M1 or M3 mutations showed normal morphology, whereas the microvesicles (MVs) (right column) showed low electric density (indicated with black arrows), suggesting that mutations of SEC23B lead to deficiency of proteins in MVs. Then we extracted the contents in MVs for MS analysis, and found that the majority of proteins in MVs of mutant cells were decreased compared with MVs of WT cell (Supplementary Table 6 and Supplementary Fig. 3b), consistent with the observation of TEM. GO analysis of proteins down-regulated in MVs of mutant cells revealed over-representation of cell adhesion and extracellular matrix proteins (Fig. 5f, g), indicating that mutant SEC23B inhibits the transport of cell adhesion proteins.

### Abnormal distribution of EPCAM and CD9 in cells with mutant SEC23B

**Fig. 6:**
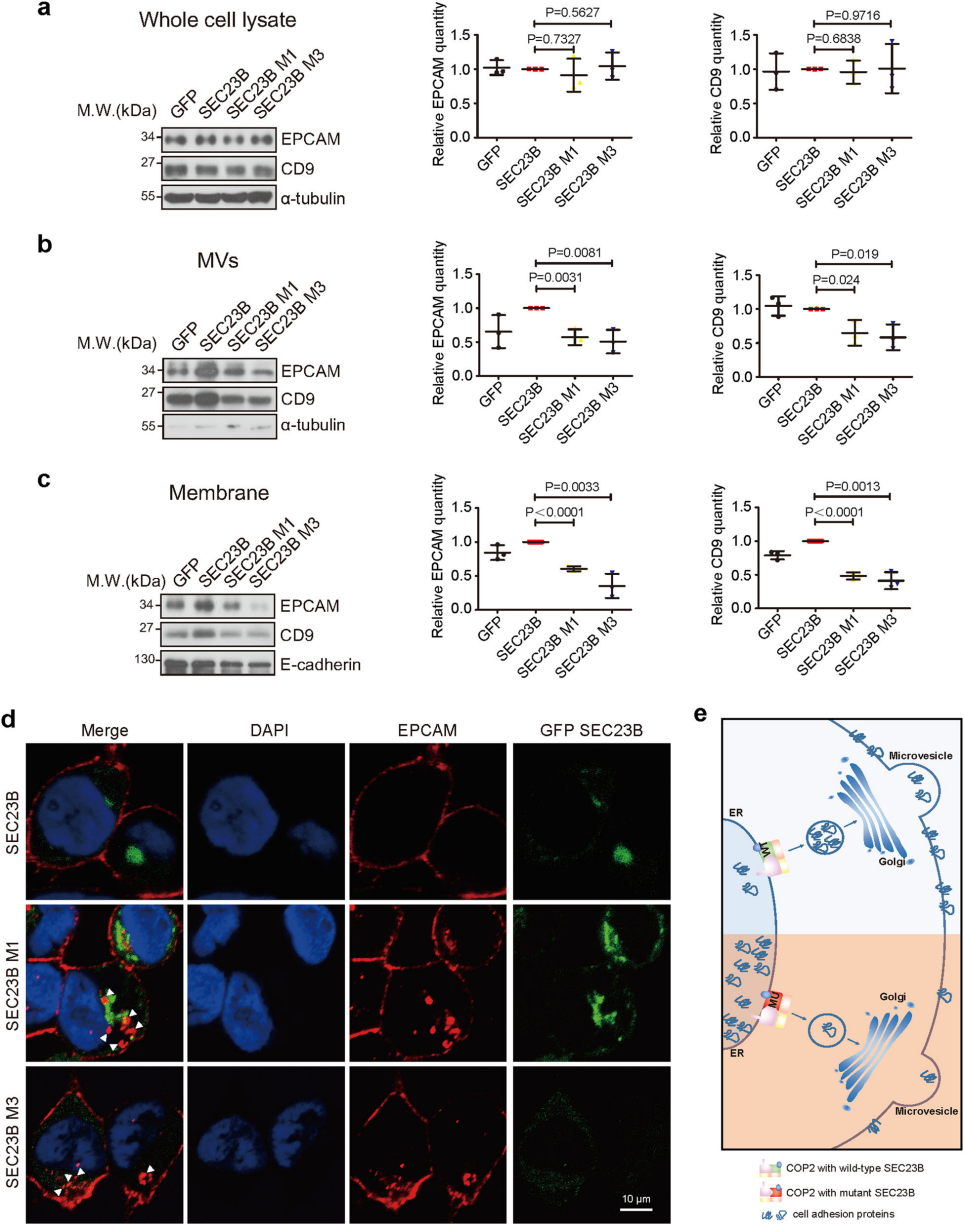
EPCAM and CD9 distribute abnormally in cells with SEC23B mutations. **a** Protein levels of EPCAM and CD9 in whole cell lysate of SW480 cells. The relative protein quantities of EPCAM and CD9 are shown in middle and right row, respectively (*T* test). α-Tubulin was used as loading control. **b** Protein levels of EPCAM and CD9 in MVs of SW480 cells. The relative protein quantities of EPCAM and CD9 are shown in middle and right row, respectively (T test). There are less EPCAM and CD9 in MVs of cells with mutant SEC23B than WT cells (lanes 3 and 4 vs. lane 2). α-Tubulin was used as loading control. **c** Protein levels of EPCAM and CD9 in the membrane of SW480 cells. The relative protein quantities of EPCAM and CD9 are shown in middle and right row, respectively (T test). There are less EPCAM and CD9 in the membrane of cells with mutant SEC23B than WT cells (lanes 3 and 4 vs. lane 2). E-cadherin was used as an indicator for cell membrane and loading control. **d** Distribution of EPCAM (red) in cells with WT or mutant SEC23B (green) measured by IF. Cell nuclei are shown with DAPI staining (blue). White arrows indicate the positions of EPCAM in the cytoplasm. Scale bars: 10 μm. **e** Graphical abstract illustrating the mechanism by which mutant SEC23B promotes tumor metastasis. WT SEC23B protein helps with the transport of cell adhesion proteins. Mutations of SEC23B disturb the protein transport function and suppress the distribution of cell adhesion proteins, thereby promoting tumor metastasis. “WT” represents WT SEC23B. “Mu” represents mutant SEC23B.

To validate the impaired cell adhesion protein transport capability caused by SEC23B mutants, we examined the distribution of EPCAM and CD9, which were found to be significantly decreased in the MVs of mutant cells (Supplementary Table 6). First, we detected the expression of EPCAM and CD9 in whole cell lysate of SW480 cells (Fig. 6a), HCT116 cells (Supplementary Fig. 4a) and DLD1 cells (Supplementary Fig. 4b). The result showed no obvious difference in EPCAM or CD9 expression between cells, demonstrating that SEC23B mutants do not influence the expression of EPCAM or CD9. Next, the EPCAM and CD9 expression in MVs were detected, and the result showed remarkable decrease of EPCAM and CD9 distribution in the MVs of cells with mutant SEC23B (Fig. 6b, Supplementary Fig. 4c, d), consistent with the MS analysis. Besides, the distribution of EPCAM and CD9 on the cell membrane were also found to be inhibited by SEC23B mutants (Fig. 6c, Supplementary Fig. 4e, f).

IF was used to confirm the abnormal distribution of EPCAM. EPCAM in cells with WT SEC23B located primarily on the cell membrane (Fig. 6d, upper row). However, cytoplasmic localization of EPCAM was found in a large number of cells with M1 (Fig. 6d, middle row) and M3 (Fig. 6d, bottom row) mutations of SEC23B. The cytoplasmic retention of EPCAM in SEC23B mutant cells suggests that EPCAM transport is inhibited by SEC23B mutations. Collectively, these results argue that M1 and M3 mutations impair the protein transport function of SEC23B, thereby inhibiting the distribution of extracellular proteins, such as EPCAM and CD9 (Fig. 6e).

### Mutations of SEC23B attenuate cell adhesion and augment cell mobility

**Fig. 7:**
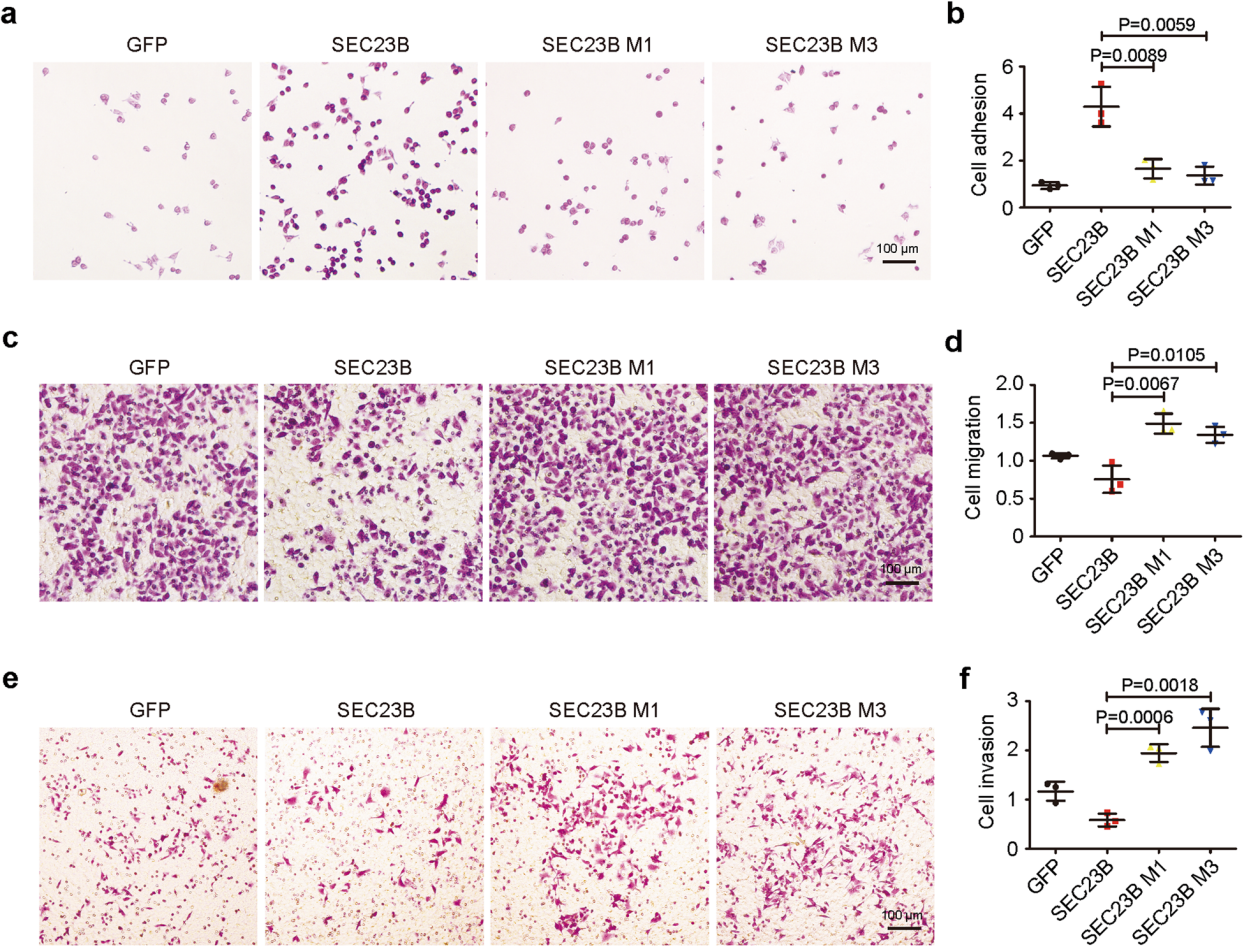
Mutations of SEC23B attenuate cell adhesion and augment cell mobility. **a**, **b** Representative images (**a**) and statistical analysis (three independent experiments) (**b**) of cell adhesion assay. M1 and M3 mutations of SEC23B decrease cell adhesion capacity (rows 3 and 4 vs. row 2, *T* test). Scale bars: 100 μm. **c**, **d** Representative images (**c**) and statistical analysis (three independent experiments) (**d**) of the cell migration assay. M1 and M3 mutations of SEC23B augment cell migration (rows 3 and 4 vs. row 2, T test). Scale bars: 100 μm. **e**, **f** Representative images (**e**) and statistical analysis (three independent experiments) (**f**) of cell invasion assay. M1 and M3 mutations of SEC23B augment cell invasion (rows 3 and 4 vs. row 2, *T* test). Scale bars: 100 μm.

To assess the metastatic potential of SEC23B mutant cells, we evaluated the proliferation rate of cells with mutant SEC23B, and found no significant difference between these cells (Supplementary Fig. 5). This result indicates that the metastases in patients were unlikely due to overgrowth of the primary tumor. Meanwhile, stronger adhesion capacity of cells with mutant SEC23B was found in Fig. 7a, b. Then we monitored the migration and invasion capacity and found increased migration (Fig. 7c, d) and invasion (Fig. 7e, f) capacities in cells with mutant SEC23B. These findings indicate that SEC23B mutations promote cell mobility and invasiveness.

### SEC23B mutations promote metastasis in vivo

**Fig. 8:**
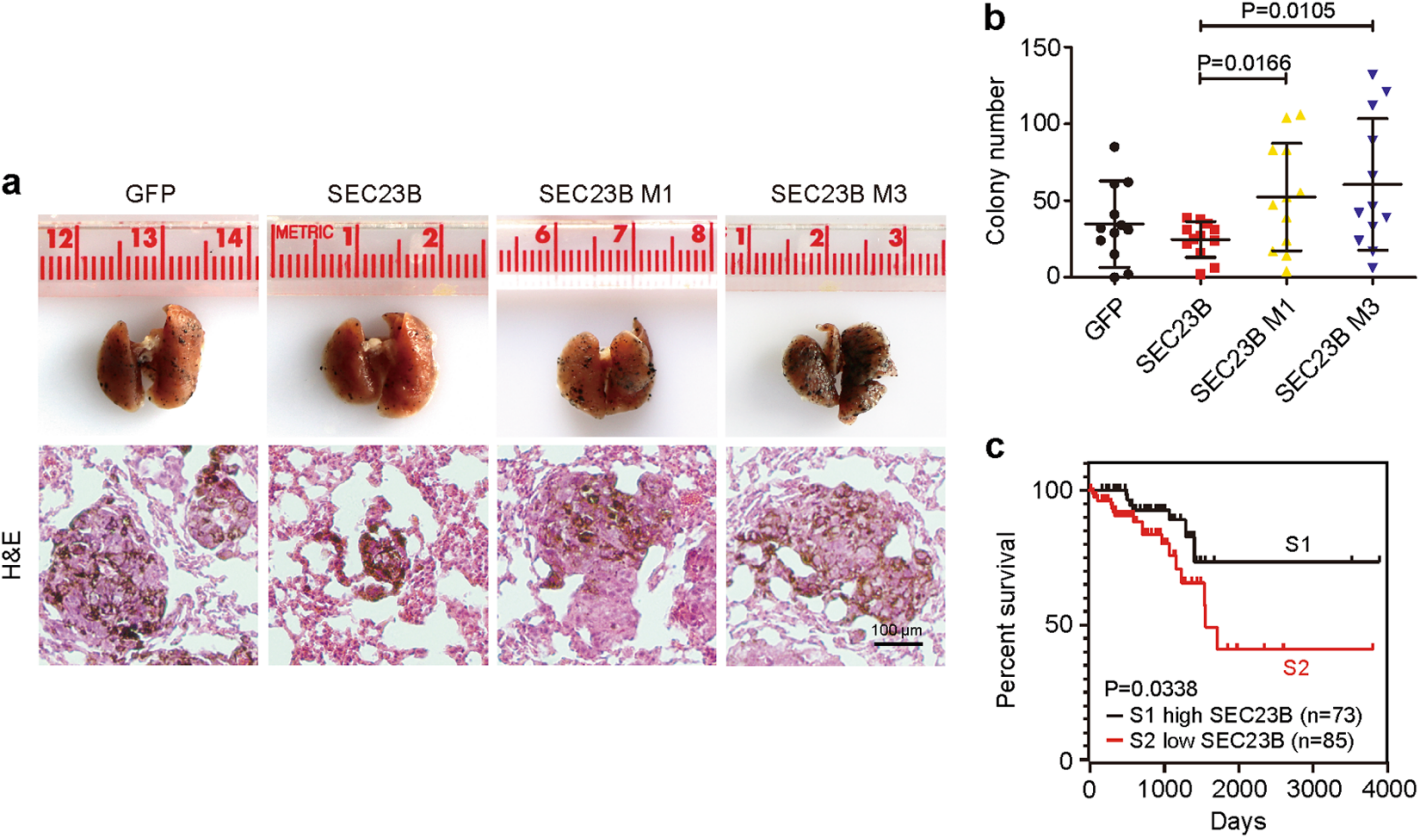
SEC23B Mutations promote metastasis in animal model and clinical data. **a** Representative photos (upper row) and H&E staining images (bottom row) of B16 metastasis model. The B16 cells were expressed with empty plasmid, WT, M1 or M3 mutations of SEC23B, respectively. Lung of mice injected with mutant B16 exhibits more metastases (columns 3 and 4 vs. column 2). Scale bars: 100 μm. **b** Statistical analysis of B16 cell lung metastases. Metastatic sites larger than 0.5 cm in diameter were counted. B16 cells with mutant SEC23B show more metastatic sites in lung (columns 3 and 4 vs. column 2, *T* test). **c** The survival rate of rectal cancer patients according to SEC23B expression. S1 (black) represents patients with higher levels of SEC23B expression, *n* = 73. S2 (red) represents patients with lower levels of SEC23B expression, *n* = 85. Patients with lower levels of SEC23B live shorter than those with higher levels of SEC23B (red line vs. black line). (Log-rank test).

To further investigate the pro-metastatic function of SEC23B mutations in vivo, we generated an experimental metastasis model with B16 cells expressing WT or mutant SEC23B (Fig. 8a, upper row). The histopathology of metastatic lesions was observed with hematoxylin and eosin (H&E) staining (Fig. 8a, bottom row). The number of lung metastases was quantified, and mice injected with mutant SEC23B cells showed more metastases than those injected with WT SEC23B (Fig. 8b), suggesting that SEC23B mutations promote tumor metastasis in vivo.

### Prognostic significance of SEC23B in CRC

To obtain insights into the impact of SEC23B mutations clinically, we further examined the significance of SEC23B on CRC progression, and found that patients with lower level of SEC23B tended to have shorter survival (Fig. 8c), indicating that low level of SEC23B accelerate tumor progression. Moreover, SEC23B expression was also found to be negatively correlated with tumor malignancy in both colon cancer (Supplementary Fig. 6a) and rectal cancer (Supplementary Fig. 6b). In conclusion, SEC23B expression may serve as a promising prognostic marker for CRC progression and outcome.

## Discussion

Metastasis has long known to be derived from sub-clones of the primary tumor with little alterations, and recent discoveries using single-cell sequencing technique and evolution analysis further confirmed this notion^36,37^. Therefore, analysis of the genetic features of primary tumors would permit the discovery of driver mutations for metastasis. Our study compared the whole-exome sequencing results of primary tumors from patients with or without MLM to explore novel genetic features and seek for predictors for CRC metastasis. To be noted, the somatic mutation burden varied among the tumors. After checking the microsatellite instability (MSI) status and DNA polymerase epsilon (POLE) status (Supplementary Table 7), we found no difference in MSI status or POLE mutation rates between the MLM group and the control group, suggesting that mutation burden is not the major reason for tumor metastasis.

By screening and bioinformatics analysis of the mutations in sequencing results, we identified a list of genes with somatic mutations specifically in the MLM group. In this article, we selected the gene *SEC23B* for functional analysis, and we expect that investigation of the other eight genes in this pool may yield other metastatic mechanisms of interest.

Previous reports focus on the genetic mutations of *SEC23B*^16,17^. However, studies on somatic mutations of *SEC23B* seem lacking. To our knowledge, our study is the first to focus on the mechanism by which *SEC23B* mutation promotes CRC metastasis. We identified five *SEC23B* mutations in our MLM cases, and each of these mutations was located in a site that is conserved across species, indicating that mutations of these sites may be pathogenic.

MVs have long known to participant in the tumor metastasis process^38^. However, most studies focus on the metastatic-promoting functions of MVs, little is known about the anti-metastatic roles of MVs. Our study highlights the metastatic-inhibiting function of MVs. In this study, contents in MVs, as exemplified by EPCAM and CD9, modulate cell adhesion and mobility. Loss of EPCAM and CD9 in MVs due to SEC23B mutations augment cell mobility and promote metastasis.

It is worth to mention that in order to validate the SEC23B mutation rate, we also identified 1 deletion mutation in 5 CRC patients with metastasis, and 1 missense mutation in 48 patients without metastasis, making the mutation rate in these two groups to be 20% and 2% correspondingly. This suggests that SEC23B mutation may serve as a potential marker for CRC metastasis. However, more samples are needed to confirm the accurate mutation rate of SEC23B in CRC metastasis.

In conclusion, our data identify specific somatic mutations of *SEC23B* in CRC patients with MLM. The C649T, C1467G and T488C + G791A + G2153A mutations are loss-of-function mutations, which inhibit the transport of extracellular matrix proteins, such as EPCAM and CD9, thereby attenuating cell adhesion and promoting tumor metastasis. Our study demonstrates the function of protein transport in CRC liver metastasis, and we propose that SEC23B is a suppressor of CRC metastasis.

## Supplementary information

Supplementary Figure legends

Supplementary Fig. 1

Supplementary Fig. 2

Supplementary Fig. 3

Supplementary Fig. 4

Supplementary Fig. 5

Supplementary Fig. 6

Supplementary Fig. 7

Supplementary Fig. 8

Supplementary Fig. 9

Supplementary Fig. 10

Supplementary Fig. 11

Supplementary Fig. 12

Supplementary Fig. 13

Supplementary Fig. 14

Supplementary Fig. 15

Supplementary Table 1

Supplementary Table 2

Supplementary Table 3

Supplementary Table 4

Supplementary Table 5

Supplementary Table 6

Supplementary Table 7

## Data Availability

The whole-exome sequencing data have been deposited in the National Center for Biotechnology Information (NCBI) Bioproject under accession number PRJNA588158. The Cancer Genome Atlas (TCGA) data were downloaded from cBioportal^39,40^ (http://www.cbioportal.org/), by searching Colorectal Adenocarcinoma^4^. The survival data of patients with different levels of SEC23B expression are available on OncoLnc^41^ (http://www.oncolnc.org/). The SEC23B expression in different stages of colon cancer and rectal cancer were downloaded from UALCAN^42^ (http://ualcan.path.uab.edu/).

## Code availability

The code for whole-exome sequencing analysis are available from the corresponding author on reasonable request.
