# Supplementary material for: Mutations in the coat complex II component SEC23B promote colorectal cancer metastasis
Source: Cell Death Dis. 2020 Mar 2;11(3):157. doi: 10.1038/s41419-020-2358-7 (PMC7052170; doi:10.1038/s41419-020-2358-7)
Supplement: Supplementary file 1 — Supplementary Figure legends [file 41419_2020_2358_MOESM1_ESM.docx]

**Supplementary Figure Legends**

**Supplementary Fig. 1** SEC23B monoclonal antibody validation and cell proliferation analysis.

**a** Validation of a monoclonal antibody against SEC23B generated in our laboratory. Lane 1-SW480 cell. Lane 2-SW480 cells heterozygous knockout of SEC23B. Lane 3-SW480 cells overexpressing pEGFP N1 SEC23B. Lane 4-SW480 cells overexpressing pCMV 2B SEC23B.

**b** SEC23B knockout decreases cell proliferation rates. Viability of cells with normal (black line) or reduced level of SEC23B (red line) was measured by MTT assay over four days. (Two-way ANOVA, ns, p >0.05).

**Supplementary Fig. 2** Analysis of protein production rate of wild-type and mutant SEC23B.

SW480 cells over-expressing wild-type or mutant SEC23B were treated with 100 µg/ml MG132, collected at 0, 12 or 24 hours and immunoblotted with antibodies against GFP and α-Tubulin.

**Supplementary Fig. 3** Heatmap analysis of the MS results.

**a** Heatmap of pulldown analysis results of wild-type or mutant SEC23B. The color yellow represents higher level of binding affinity. The color blue represents lower level of binding affinity. Mutations of SEC23B show decreased binding affinity with a large number of proteins than WT SEC23B (lanes 2 and 3 vs. lane 1).

**b** Heatmap of proteins in the microvesicles of cells with WT and mutant SEC23B. The color yellow represents higher level of protein quantity. The color blue represents lower level of protein quantity. M1 and M3 mutations of SEC23B decrease the contents in the microvesicles (lanes 2 and 3 vs. lane 1).

**Supplementary Fig. 4** EPCAM and CD9 distribute abnormally in cells with SEC23B mutations.

**a, b** Protein levels of EPCAM and CD9 in whole cell lysate of HCT116 (**a**), and DLD1 (**b**) cells with GFP-tagged WT, M1 or M3 mutation of SEC23B. The relative protein quantities of EPCAM and CD9 are shown in the middle and right row, respectively. (T test). α-Tubulin was used as loading control.

**c, d** Protein levels of EPCAM and CD9 in the MVs of HCT116 (**c**), and DLD1 (**d**) cells. The relative protein quantities of EPCAM and CD9 are shown in the middle and right row, respectively. (T test). There are less EPCAM and CD9 in the MVs of cells with mutant SEC23B than the wild-type cell (lanes 3 and 4 vs. lane 2). α-Tubulin was used as loading control.

**e, f** Protein levels of EPCAM and CD9 in the membrane of HCT116 (**e**), and DLD1 (**f**) cells. The relative protein quantities of EPCAM and CD9 are shown in the middle and right row, respectively. (T test). There are less EPCAM and CD9 in the membrane of cells with mutant SEC23B than the wild-type cell (lanes 3 and 4 vs. lane 2). E-cadherin was used as an indicator for cell membrane and loading control.

**Supplementary Fig. 5** Proliferation rate of cells with wild-type or mutant SEC23B.

Proliferation rate of cells with GFP, GFP-tagged wild-type or mutant SEC23B. Cell viability was measured by MTT assay over three days. There is no significant difference in survival rate between cells with wild-type or mutant SEC23B (yellow line and blue line vs. red line). (Two-way ANOVA, ns, p >0.05).

**Supplementary Fig. 6** SEC23B expression level negatively correlates with CRC malignancy.

**a, b** The expression of SEC23B in different stages of colon cancer (**a**) and rectal cancer (**b**). Data were acquired from UALCAN.

**Supplementary Fig. 7** Raw western blot data of Fig. 3a.

**Supplementary Fig. 8** Raw western blot data of Fig. 3c.

**Supplementary Fig. 9** Raw western blot data of Fig. 3e.

**Supplementary Fig. 10** Raw western blot data of Fig. 4a.

**Supplementary Fig. 11** Raw western blot data of GFP-SEC23B in Fig. 4b.

**Supplementary Fig. 12** Raw western blot data of GAPDH in Fig. 4b.

**Supplementary Fig. 13** Raw western blot data of Fig. 6a.

**Supplementary Fig. 14** Raw western blot data of Fig. 6b.

**Supplementary Fig. 15** Raw western blot data of Fig. 6c.

**Supplementary Table 1** Nine genes with functional alterations identified in MLM patients.

**Supplementary Table 2** Conservation of mutations in SEC23B.

^1^Positions refer to GenBank transcript NM_032985.5.

^2^PhyloP scores range between -14 and +6; conserved sites have positive scores.

^3^PhastCons scores range between 0 and 1; values closer to 1 have a higher probability of nucleotide conservation.

**Supplementary Table 3** Predictive analysis of SEC23B mutations in patient S1, S2 and S7 performed by Polyphen-2, and SIFT.

^1^Positions refer to GenBank transcript NM_032985.5.

^2^Catalogue of somatic mutations in cancer. NA is short for not available for evaluation; 1 represents one case found in endometrial cancer.

^3^Function prediction tool. D is short for probably damaging (>= 0.957). P is short for possibly damaging.

^4^Function prediction tool based on protein sequence conservation among homologs.

**Supplementary Table 4** Mass spectrum analysis of proteins on the membrane of cell knockout of SEC23B and the control cell.

^1^Accession refers to Uniprot number of proteins.

^2^Area refers to the quantity of proteins identified by mass spectrum analysis.

**Supplementary Table 5** List of proteins identified by mass spectrum in flag pulldown analysis of wild type or mutant SEC23B binding proteins.

^1^Accession refers to Uniprot number of proteins.

^2^Area refers to the quantity of proteins identified by mass spectrum analysis.

**Supplementary Table 6** List of proteins identified by mass spectrum in the microvesicles of cells with wild type or mutant SEC23B binding proteins.

^1^Accession refers to Uniprot number of proteins.

^2^Area refers to the quantity of proteins identified by mass spectrum analysis.

**Supplementary Table 7** Microsatellite stability status and POLE status of patient tumors in our study.

^1^Genes represents DNA mismatch repair genes. “0” represents negative expression in IHC detection. “1” represents positive expression in Immunohistochemistry (IHC) detection.

^2^Microsatellite stability status. “MSS” represents microsatellite stable. “MSI-L” means low level of microsatellite instability. “MSI-H” means high level of microsatellite instability.

^3^POLE status. “0” means no mutation in POLE gene. “1” means one mutation in POLE gene. “2” means two mutations in POLE gene.
