# Supplementary material for: Mutations in the coat complex II component SEC23B promote colorectal cancer metastasis
Source: Cell Death Dis. 2020 Mar 2;11(3):157. doi: 10.1038/s41419-020-2358-7 (PMC7052170; doi:10.1038/s41419-020-2358-7)
Supplement: Supplementary file 18 — Supplementary Table 2 [file 41419_2020_2358_MOESM18_ESM.docx]

**Supplementary Table 2** Conservation of mutations in SEC23B.

| Nucleotide alteration^1^ | T488C | C649T | G791A | C1467G | G2153A |
| --- | --- | --- | --- | --- | --- |
| PhyloP^2^ | 4.638 | -0.056 | 4.231 | 0.252 | 5.351 |
| PhastCons^3^ | 1 | 0.031 | 1 | 0.998 | 1 |
