# Supplementary material for: Mutations in the coat complex II component SEC23B promote colorectal cancer metastasis
Source: Cell Death Dis. 2020 Mar 2;11(3):157. doi: 10.1038/s41419-020-2358-7 (PMC7052170; doi:10.1038/s41419-020-2358-7)
Supplement: Supplementary file 19 — Supplementary Table 3 [file 41419_2020_2358_MOESM19_ESM.docx]

**Supplementary Table 3** Predictive analysis of SEC23B mutations in patient S1, S2 and S7 performed by Polyphen-2, and SIFT.

| Nucleotide alteration^1^ | T488C | C649T | G791A | C1467G | G2153A |
| --- | --- | --- | --- | --- | --- |
| Amino acid alteration | L163P | R217X | R264Q | H489Q | R718Q |
| COSMIC^2^ | NA | NA | 1 | NA | 1 |
| PolyPhen 2 HDIV^3^ | D | - | D | P (0.774) | D |
| PolyPhen 2 Hvar^3^ | D | - | D | P (0.566) | D |
| SIFT^4^ | D | - | D | D | D |
