# Supplementary material for: Mutations in the coat complex II component SEC23B promote colorectal cancer metastasis
Source: Cell Death Dis. 2020 Mar 2;11(3):157. doi: 10.1038/s41419-020-2358-7 (PMC7052170; doi:10.1038/s41419-020-2358-7)
Supplement: Supplementary file 23 — Supplementary Table 7 [file 41419_2020_2358_MOESM23_ESM.docx]

**Supplementary Table 7** Microsatellite stability status and POLE status of patient tumors in our study.

| Patient number | Genes^1^ | | | | MSI status^2^ | POLE status^3^ |
| --- | --- | --- | --- | --- | --- | --- |
|  | MLH1 | MSH2 | MSH6 | PMS2 |  |  |
| S1 | 1 | 1 | 1 | 1 | MSS | 0 |
| S2 | 1 | 1 | 1 | 1 | MSS | 2 |
| S3 | 1 | 1 | 1 | 1 | MSS | 0 |
| S4 | 1 | 1 | 0 | 1 | MSI-L | 0 |
| S6 | 0 | 1 | 1 | 0 | MSI-H | 0 |
| S7 | 1 | 1 | 0 | 1 | MSI-L | 0 |
| S8 | 0 | 1 | 1 | 1 | MSI-L | 0 |
| S9 | 1 | 1 | 0 | 1 | MSI-L | 1 |
| S10 | 0 | 1 | 1 | 0 | MSI-H | 0 |
| S11 | 1 | 1 | 1 | 1 | MSS | 0 |
| S12 | 0 | 1 | 1 | 1 | MSI-L | 0 |
| S13 | 1 | 1 | 1 | 1 | MSS | 0 |
| S14 | 0 | 1 | 1 | 0 | MSI-H | 1 |
| S15 | 1 | 0 | 0 | 1 | MSI-H | 1 |
| S16 | 0 | 1 | 1 | 0 | MSI-H | 0 |
| S17 | 1 | 1 | 1 | 1 | MSS | 0 |
| S18 | 1 | 1 | 1 | 1 | MSS | 0 |
| S19 | 1 | 1 | 1 | 1 | MSS | 0 |
| S20 | 1 | 1 | 1 | 1 | MSS | 0 |
| S21 | 1 | 1 | 1 | 1 | MSS | 0 |
